# Digital health competencies in medical school education: a scoping review and Delphi method study

**DOI:** 10.1186/s12909-022-03163-7

**Published:** 2022-02-26

**Authors:** Mark P. Khurana, Daniel E. Raaschou-Pedersen, Jørgen Kurtzhals, Jakob E. Bardram, Sisse R. Ostrowski, Johan S. Bundgaard

**Affiliations:** 1 Department of Clinical Medicine, Faculty of Health and Medical Sciences, University of Copenhagen, Blegdamsvej 3B, 2200 Copenhagen, Denmark; 2 Department of Immunology and Microbiology, Faculty of Health and Medical Sciences, University of Copenhagen, Copenhagen, Denmark; 3 Department of Clinical Microbiology, Rigshospitalet, Copenhagen University Hospital, Copenhagen, Denmark; 4 Department of Health Technology, Technical University of Denmark, Lyngby, Denmark; 5 Department of Clinical Immunology, Rigshospitalet, Copenhagen University Hospital, Copenhagen, Denmark; 6 Department of Cardiology, Rigshospitalet, Copenhagen University Hospital, Copenhagen, Denmark

**Keywords:** Digital health, Medical education, Delphi method

## Abstract

**Introduction:**

In order to fulfill the enormous potential of digital health in the healthcare sector, digital health must become an integrated part of medical education. We aimed to investigate which knowledge, skills and attitudes should be included in a digital health curriculum for medical students through a scoping review and Delphi method study.

**Methods:**

We conducted a scoping review of the literature on digital health relevant for medical education. Key topics were split into three sub-categories: knowledge (facts, concepts, and information), skills (ability to carry out tasks) and attitudes (ways of thinking or feeling). Thereafter, we used a modified Delphi method where experts rated digital health topics over two rounds based on whether topics should be included in the curriculum for medical students on a scale from 1 (strongly disagree) to 5 (strongly agree). A predefined cut-off of ≥4 was used to identify topics that were critical to include in a digital health curriculum for medical students.

**Results:**

The scoping review resulted in a total of 113 included articles, with 65 relevant topics extracted and included in the questionnaire. The topics were rated by 18 experts, all of which completed both questionnaire rounds. A total of 40 (62%) topics across all three sub-categories met the predefined rating cut-off value of ≥4.

**Conclusion:**

An expert panel identified 40 important digital health topics within knowledge, skills, and attitudes for medical students to be taught. These can help guide medical educators in the development of future digital health curricula.

**Supplementary Information:**

The online version contains supplementary material available at 10.1186/s12909-022-03163-7.

## Background

Digital health, an umbrella term broadly defined as the use of digital technologies for health, [1] has progressively become an integrated part of healthcare practice. Spanning a wide range of fields, including electronic health records (EHRs), telehealth, mobile and wearable health technology (mHealth), and artificial intelligence (AI), digital health has been heralded as a means by which to increase the delivery of and access to healthcare [2, 3]. AI is already being used increasingly in clinical practice, and has been validated for e.g. image recognition, tumor identification, respiratory syndrome differentiation, etc. [4–6] Smartphone apps and other mHealth technologies have also been introduced as screening and monitoring tools for a wide range of ailments, including eye health, mental health, and a range of chronic diseases [7–9]. As such, digital health technology is playing an increasingly important role in clinical practice.

The Covid-19 pandemic has accelerated the digital health trend, specifically with regards to telehealth and telemonitoring of patients unable to access their typical healthcare providers [10]. While the implementation of digital health has so far been highly heterogeneous, the potential of digital health is enormous [11]. However, the fulfillment of this potential is dependent upon future healthcare workforces being competent and comfortable with its use [12–14].

Doctors are particularly critical in the process of implementing digital health in the healthcare sector. Given the role of doctors in guiding patient treatment, digital health is unlikely to be routinely implemented unless they are engaged in its use. For this to happen, digital health must become an integrated part of medical education. While there have been calls for its inclusion into medical curricula, digital health is not routinely included in medical education - although digital health courses prior to and during medical school do exist [15–18]. In order for a systematic integration to occur, consensus regarding the topics to include, as well as those not to include, in such a curriculum need to be established. To contribute to this knowledge base, we conducted a Delphi study among Danish digital health experts to define the most important topics to be included in a digital health curriculum for medical students.

## Method

### Delphi method and scoping review

A modified Delphi method was used to identify key topics to include in a digital health curriculum for medical students. The modified Delphi method is a common method of achieving consensus within a group that is based on six stages: agreeing on a research question, an initial literature search, the development of a questionnaire, multiple iterative rounds of questionnaires, feedback to participants between rounds and consequently a summary of the findings [19, 20]. The four key features of consensus methods are anonymity, iteration, statistical group response and controlled feedback [21]. The benefits of the Delphi method include the large number of potential participants, anonymity in the expert panel group (avoiding undue intra-group influence between participants) and that questionnaires can be filled remotely [22].

In order to identify relevant topics for inclusion in the questionnaire for the expert panel, we conducted a scoping review based on the methodology developed by Arksey and O’Malley [23]. The scoping review was based on the following research question: “*What knowledge, skills and attitudes (KSAs) within digital health are essential for future doctors?*”. Relevant information pertaining to the research question was found through MEDLINE with the assistance of a medical librarian. We searched the following MeSH terms: digital health, biohack*, biomedical engineering, biomedical technology, medical informatics, telemedicine*, electronic health record*, wearable electronic devices*, algorithm*, medical student*, and medical education*. The search string used was: ((digital health*[Text Word]) OR (biohack*[Text Word]) OR (biomedical engineering*[MeSH Terms]) OR (biomedical technology*[MeSH Terms]) OR (medical informatics*[MeSH Terms]) OR (telemedicine*[MeSH Terms]) OR (electronic health record*[MeSH Terms])) OR (wearable electronic devices*[MeSH Terms]) OR (algorithm**[MeSH Terms]) AND ((medical student*[MeSH Terms]) OR (medical education*[MeSH Terms])) AND (2015:2020[pdat]).

**Fig. 1:**
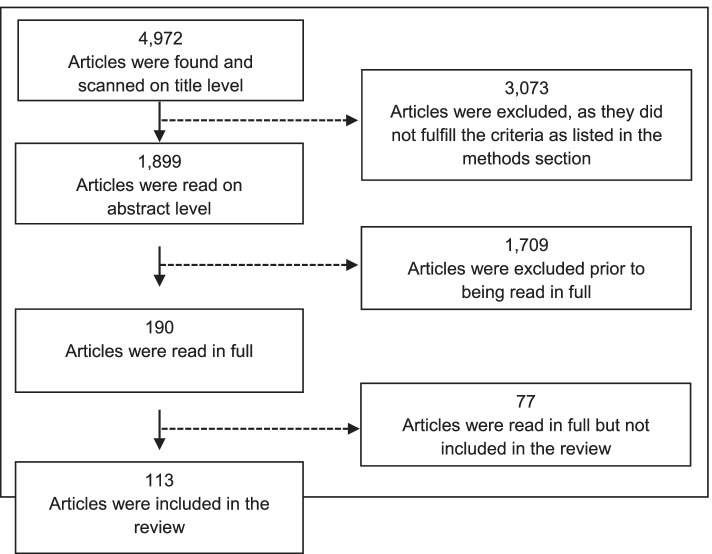
Flowchart for articles included in the scoping review

Multiple reviewers (DRP, MPK, JSB) reviewed the literature, with each article being reviewed by two reviewers. In the first round, 4972 titles were scanned. Those relevant to either medical education or digital health that were published between 2015 and 2020 in the English language were included. Hereafter, 1899 abstracts from relevant articles were scanned to identify those that were directly relevant to both digital health and medical education. 190 articles were read in full and 113 were included in the final review (Fig. 1). Topics were extracted if they were deemed to fall within the broad definition of digital health, namely the use of digital technologies for health, using the mutually exclusive and collectively exhaustive principle [1].

### Expert panel

Potential participants for the Delphi expert panel were identified through authors and references from the scoping review (8 experts), referrals from academic directors (3 experts), and referrals from academic community members (7 experts). To ensure diversity and representativeness in the participant group, we utilized a purposive sampling methodology. We selected participants to achieve diversity across several domains, including educational background (e.g., medical professionals, healthcare technology experts, social scientists), medical specialization, geography, seniority, and institutional settings. Convenience sampling was used until sources of potential experts were exhausted. All participants were based in Denmark and were employed by public institutions. We defined an expert as an individual who satisfied one of the following two criteria: 1) works professionally with digital health research/implementation or 2) is active in the development of medical education curricula with knowledge of digital health. A total of 35 participants were invited by e-mail to participate. Participants were informed of the purpose of the study with full anonymity between experts. Participants were not offered any remuneration for completion of the surveys.

### Questionnaire development and completion

The primary method for developing the questionnaire was the inclusion of topics identified during the scoping review; several other reports not published as articles were also reviewed for digital health topics (Additional file 1). Key topics were collated into a master list. The keywords were split into three commonly utilized sub-categories: knowledge, skills, and attitudes. Knowledge is defined as the understanding of facts, concepts, and information. Skills are defined as abilities to carry out particular tasks. Attitudes are settled ways of thinking or feeling about something. A total of 63 items were included in the first-round questionnaire, and 65 items were included in the final round questionnaire since experts could suggest additional topics following the first questionnaire round.

Questionnaires were developed using Google Forms, and invitations were sent to participants by email. Experts rated the topics in the questionnaire based on whether they believed the topic should be integrated into a digital health curriculum for medical students. Topics were rated from 1 to 5 (1 = Strongly disagree, 2 = Disagree, 3 = Neutral, 4 = Agree and 5 = Strongly agree). Additional topics not included in the first round could be suggested by participants to be added in the second round. After the first round, participants were provided average ratings for each topic from the previous round. We completed two questionnaire rounds and used a predefined rating cut-off of ≥4 to identify topics that were critical to include in a digital health curriculum for medical students.

## Results

### Characteristics of the expert panel

**Fig. 2:**
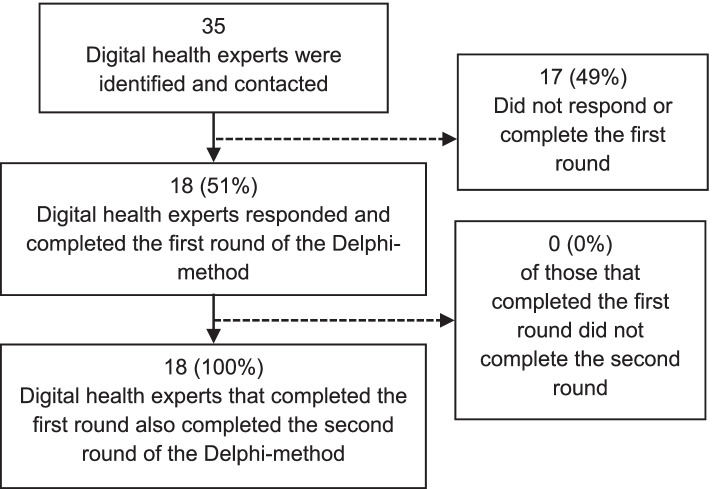
Flowchart for inclusion of digital health experts

In total, of the 35 invited digital health experts, 18 (51%) completed both the first and second rounds of questionnaires (Fig. 2). All experts (18/18) that completed the first round subsequently also completed the second round. 12 of the 18 (67%) participants were male, 7 of the 18 experts were physicians (39%) (Additional file 2) and 77% of the participants had a PhD or a higher doctorate, with roles divided between directors, head of departments, vice-deans, associate professors, and a civil servant (Table 1).

**Table 1:** Demographic characteristics of the expert panel

Characteristics	Expert panel (*n* = 18) Full completion^a^
Gender – n (%)	
Male	12 (67)
Female	6 (33)
Highest attained degree – n (%)	
High School	1 (6)
Master’s degree	3 (17)
PhD	8 (44)
Higher doctorate	6 (33)
Current role – n (%)^b^	
Director	3 (17)
Head of department	3 (17)
Vice-dean	1 (6)
Associate professor^c^	7 (39)
Professor	5 (28)
Civil servant	1 (6)

^a^Full completion is defined as an expert that responded to both rounds

^b^Three experts held multiple titles and are therefore counted twice

^c^Associate professor includes two experts that are now associate professor emeritus

### Rating of topics

**Fig. 3:**
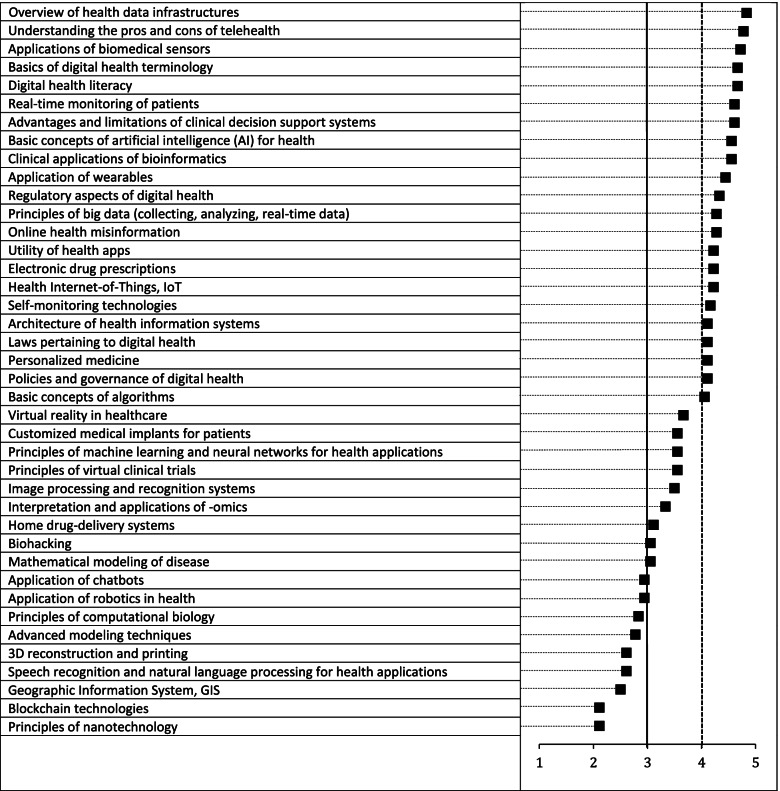
Average ratings by the expert panel for digital health knowledge

Of the knowledge topics identified during the scoping review, 22 met the pre-defined cut-off of ≥4 in the second round of questionnaires (Fig. 3). Nine topics were ranked between 3 and 4, with nine rated < 3. Two new knowledge topics were included in the second round after being suggested by participants in the first round: “*Principles of virtual clinical trials (e.g., clinical trials run remotely)*” and “*Advantages and limitations of clinical decision support systems*”. Broad introductions to topics, such as health data infrastructures, digital health terminology and basic concepts of artificial intelligence were rated highly, whereas highly technical knowledge such as mathematical modeling, robotics and 3D reconstruction and printing were ranked lower. A full list of topics with average ratings, median values, and inter-quartile ranges (IQR) can be found in Additional file 3.

**Fig. 4:**
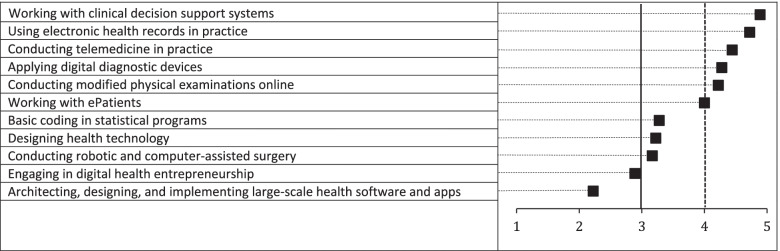
Average ratings by the expert panel for digital health skills

Among the skill topics included, five met the pre-defined cut-off of ≥4 in the second round of questionnaires (Fig. 4). Four topics were ranked between 3 and 4, with two rated < 3. No new topics were added in the second round. The three highest rated topics were “*Working with clinical decision support systems*”, “*Using electronic health records in practice*” and “*Conducting telemedicine in practice*”. One expert noted that *“The skills section has many interesting points where obtaining skills are maybe a high bar to set, but where an understanding of the dynamics behind is essential. E.g., the use of electronic health records - no need for practical teaching in the curriculum, they should be taught in the current programs of the department at their clinical internships. But they need a basic understanding of how EHRs work, how they exchange data with other systems and why it is challenging to make a proper EHR [ …*] *The same with designing digital health services. Of course, all doctors should not be able to do that. But they need a basic understanding of what goes on in the design, innovation, and implementation processes so they can relevantly contribute to the process of systems they are to use and promote in the future. The more they understand, the more relevant system they can demand.”*

**Fig. 5:**
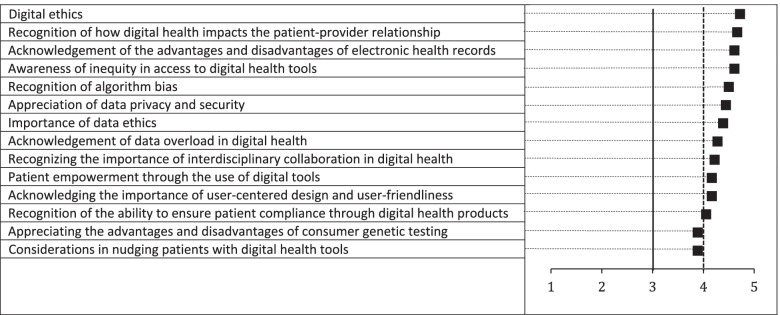
Average ratings by the expert panel for digital health attitudes

For the digital health topics on attitudes, twelve met the pre-defined cut-off of ≥4 in the second round of questionnaires (Fig. 5). Only two topics were ranked between 3 and 4. The three highest rated topics were “*Digital ethics*”, “*Recognition of how digital health impacts the patient-provider relationship*” and “*Acknowledgement of the advantages and disadvantages of electronic health records*”.

## Discussion

The digital health expert panel found that among the digital health topics identified in the medical literature, approximately half should be included in the medical curriculum. Conversely, they believed a large proportion of digital health topics within knowledge, skills, and attitudes are currently not relevant enough for future doctors to learn.

Interestingly, the proportion of topics that were important to include was highest in the attitudes section and approximately equal for the knowledge and skills sections, whereas absolute numbers were highest for knowledge (22 topics) followed by attitudes (12 topics) and skills (6 topics). Generally, the results suggested that attitudes towards digital health, and a basic understanding of digital health’s uses and limitations, were significantly more important than practical skills within digital health. This implies that collaborative skills are likely to be crucial in maximizing the potential of digital health, since physicians must be able to work in interdisciplinary environments with other specialists that have digital health skills not expected to be possessed by physicians themselves. This distinction highlights the panel’s view that the role of doctors is to understand the applications of digital health without necessarily developing solutions themselves. The higher absolute number of topics within knowledge and attitudes compared to skills identified during the scoping review further supports this insight. In addition, this is evident within each knowledge, skills, and attitudes section, where highly technical competencies were considered less important than digital health tools relating to clinical practice, such as competency with the use of EHRs, clinical decision support tools, and telemedicine solutions.

Some medical schools have implemented digital health courses, although their integration thus far has been heterogenous. In a recent scoping review of digital health courses in medical schools, the authors found that the majority of courses were elective and mainly focused on medical informatics [24]. They are thus not routinely integrated into the curriculum and tend to focus on narrow topics within digital health. In Germany, following the implementation of a 3-week curricular module on digital health at a medical school, the module was well-received by both faculty and students, both of which highlighted the importance of digital health for clinical care and its underrepresentation in the curriculum [25]. From an implementation perspective, however, there are justifiable concerns regarding the already packed medical curriculum, with fears of information overload [26, 27]. Identifying and removing outdated topics, which may follow directly from the automatization of certain clinical functions as digital health solutions become more commonplace, is therefore equally important in developing an optimal medical curriculum [28].

Several limitations should be acknowledged when interpreting the findings of the study. Firstly, the topics included from the scoping review were all articles published in English, and the expert panel was composed of experts living in Denmark. The generalizability of the results may thus primarily apply to English-speaking settings or those with similar healthcare structures and educational systems to Denmark. Another limitation of the Delphi study process is that the expert panel members may represent leading universities and are therefore not fully representative of all medical schools and institutions. Given that experts are likely to be older than non-experts, an age bias may also affect the topics that are deemed important to include, possibly skewing towards less novel but more established topics. Secondly, topics were stratified during the scoping review based on the mutually exclusive and collectively exhaustive principle, where topics were assumed not to overlap - despite the practical challenges associated with achieving this. Some topics may have overlapped, making them more challenging for the expert panel to rate, which was noted by two experts during the first questionnaire round. Additionally, ratings from the survey indicate which topics are important to prioritize in future curricula, but do not indicate how big a part of the curriculum each topic should comprise. Lastly, the field of digital health is rapidly evolving, [29] and the medical curriculum should ideally be modified to reflect this development. As such, the findings in this study represent a snapshot of current knowledge, skills, and attitudes, and should therefore be adapted regularly going forward.

The primary strength of this study is that we conducted a large scoping review prior to the Delphi process to include the most up-to-date digital health topics. We also allowed experts to include additional important topics not identified during the scoping review. The expert panel was large and diverse, with multiple clinical specialties represented as well as experts spanning from directors to civil servants to professors (Additional file 2). Additionally, the follow-up response rate was high between the first and second questionnaire rounds.

## Conclusion

An expert panel identified 40 important digital health topics within knowledge (22 topics), skills (6 topics), and attitudes (12 topics) to be taught during medical school. The average rating of each topic can be understood to represent its relative importance for inclusion in the curriculum. Given the growing role of digital tools in health, curriculum changes are needed to keep pace with this development. The insights from this study can help guide medical and digital health educators in the development of future digital health curricula.

## Supplementary Information

**Additional file 1.** Reports included for digital health topic selection.

**Additional file 2. **Demographic characteristics between invited experts and participating experts.

**Additional file 3.** Tables with average ratings, median values, and interquartile ranges for knowledge, skills, and attitudes.

## Data Availability

The datasets used and/or analyzed during the current study are available from the corresponding author on reasonable request.
