# Supplementary material for: Digital health competencies in medical school education: a scoping review and Delphi method study
Source: BMC Med Educ. 2022 Feb 26;22:129. doi: 10.1186/s12909-022-03163-7 (PMC8881190; doi:10.1186/s12909-022-03163-7)
Supplement: Supplementary file 1 — Additional file 1. Reports included for digital health topic selection. [file 12909_2022_3163_MOESM1_ESM.docx]

**Appendix 1:** Reports included for digital health topic selection

| The Rise of the Data-Driven Physician(1) |
| --- |
| Danish Digital Health Strategy, 2018-2022(2) |
| The Australian Health Informatics Competencies Framework and Its Role in the Certified Health Informatician Australasia (CHIA) Program(3) |
| Digital Health in the Medical Curriculum: Addressing the Needs of the Future Health Workforce(4) |
| The Topol Review: Preparing the healthcare workforce to deliver the digital future(5) |

1. Stanford Medicine. The Rise of the Data-Driven Physician: Stanford Medicine 2020 Health Trends Report [Internet]. 2020. Available from: https://med.stanford.edu/news/all-news/2020/01/health-trends-report-spotlights-rise-of-data-driven-physician.html

2. Danish Ministry of Health. Digital Health Strategy 2018-2022 [Internet]. 2018. Available from: https://sundhedsdatastyrelsen.dk/-/media/sds/filer/strategi-og-projekter/strategi-digital-sundhed/digital-health-strategy-2018_2022.pdf

3. Martin-Sanchez F, Rowlands D, Schaper L, Hansen D. The Australian Health Informatics Competencies Framework and Its Role in the Certified Health Informatician Australasia (CHIA) Program. Stud Health Technol Inform [Internet]. 2017;245:783–7. Available from: http://www.ncbi.nlm.nih.gov/pubmed/29295205

4. European Medical Students’ Association. Digital Health in the Medical Curriculum:Addressing the Needs of the Future Health Workforce [Internet]. Brussels; 2019. Available from: https://www.scribd.com/document/471107407/Digital-Health-in-the-Medical-Curriculum-Addressing-the-Needs-of-the-Future-Health-Workforce-pdf

5. The Topol Review: Preparing the healthcare workforce to deliver the digital future. An independent report on behalf of the Secretary of State for Health and Social Care [Internet]. 2019. Available from: https://topol.hee.nhs.uk/wp-content/uploads/HEE-Topol-Review-2019.pdf
