# Supplementary material for: Digital health competencies in medical school education: a scoping review and Delphi method study
Source: BMC Med Educ. 2022 Feb 26;22:129. doi: 10.1186/s12909-022-03163-7 (PMC8881190; doi:10.1186/s12909-022-03163-7)
Supplement: Supplementary file 2 — Additional file 2. Demographic characteristics between invited experts and participating experts. [file 12909_2022_3163_MOESM2_ESM.docx]

**Appendix 2:** Demographic characteristics between invited experts and participating experts

| Characteristics | Participating expert panel (n=18)  Full completion* | Non-participating expert panel (n=17) |
| --- | --- | --- |
| Gender – n (%) |  |  |
| Male | 12 (67) | 13 (76) |
| Female | 6 (33) | 4 (14) |
| Highest attained degree – n (%) |  |  |
| High School | 1 (6) | 0 (0) |
| Master’s degree | 3 (17) | 6 (36) |
| PhD | 8 (44) | 9 (52) |
| Higher doctorate | 6 (33) | 2 (12) |
| Current role – n (%)** |  |  |
| Director | 3 (17) | 1 (6) |
| Head of department | 3 (17) | 2 (12) |
| Vice-dean | 1 (6) | 0 (0) |
| Associate professor*** | 7 (39) | 3 (18) |
| Professor | 5 (28) | 9 (52) |
| Civil servant | 1 (6) | 2 (12) |
| Medical Specialty – n (%)**** |  |  |
| Cardiology | 3 (43) | 3 (33) |
| Microbiology | 0 (0) | 1 (11) |
| Family medicine | 0 (0) | 2 (22) |
| Public Health | 0 (0) | 2 (22) |
| Anesthesiology | 0 (0) | 1 (11) |
| Radiology | 1 (14) | 0 (0) |
| Oncology | 1 (14) | 0 (0) |
| Hematology | 1 (14) | 0 (0) |
| Other | 1 (14) | 0 (0) |

*Full completion is defined as an expert that responded to both rounds

**Three experts held multiple titles and are therefore counted twice

***Associate professor includes two experts that are now associate professor emeritus

****Among those qualified as physicians
