# Supplementary material for: Digital health competencies in medical school education: a scoping review and Delphi method study
Source: BMC Med Educ. 2022 Feb 26;22:129. doi: 10.1186/s12909-022-03163-7 (PMC8881190; doi:10.1186/s12909-022-03163-7)
Supplement: Supplementary file 3 — Additional file 3. Tables with average ratings, median values, and interquartile ranges for knowledge, skills, and attitudes. [file 12909_2022_3163_MOESM3_ESM.docx]

**Appendix 3:** Tables with average ratings, median values, and interquartile ranges (IQR) for knowledge, skills, and attitudes

Table with the average ratings, median values, and interquartile ranges (IQR) of the topics ranked by expert panel for digital health knowledge

|  | **Average Rating** | **Median** | **IQR** |
| --- | --- | --- | --- |
| Overview of health data infrastructures (e.g. types of electronic health record systems, biobanks, patient registers, information sharing and storage) | 4.83 | 5 | 5 - 5 |
| Understanding the pros and cons of telehealth (e.g. access, lower costs, compensation for provider shortage) | 4.78 | 5 | 5 - 5 |
| Applications of biomedical sensors (e.g. in vivo chips, cardiac telemetry) | 4.72 | 5 | 4.25 - 5 |
| Basics of digital health terminology | 4.67 | 5 | 4.25 - 5 |
| Digital health literacy (ability to seek, find, understand, and appraise health information from electronic sources) | 4.67 | 5 | 4 - 5 |
| Real-time monitoring of patients (e.g. wearables, glucose monitoring - in contrast to sporadic data collection) | 4.61 | 5 | 4 - 5 |
| Advantages and limitations of clinical decision support systems | 4.61 | 5 | 4 - 5 |
| Basic concepts of artificial intelligence (AI) for health | 4.56 | 5 | 4 - 5 |
| Clinical applications of bioinformatics | 4.56 | 5 | 4 - 5 |
| Application of wearables (e.g. fitness trackers, smartwatches, smartglasses, digestables) | 4.44 | 4.5 | 4 - 5 |
| Regulatory aspects of digital health (e.g. GDPR, CE marking, FDA approval) | 4.33 | 4 | 4 - 5 |
| Principles of big data (collecting, analysing, real-time data) | 4.28 | 4 | 4 - 5 |
| Online health misinformation | 4.28 | 4 | 4 - 5 |
| Utility of health apps (e.g. menstrual period trackers, medication adherence) | 4.22 | 4 | 4 - 4.25 |
| Electronic drug prescriptions | 4.22 | 4.5 | 4 - 5 |
| Health Internet-of-Things, IoT (Physical objects embedded with interconnected sensors and software) | 4.22 | 4 | 4 - 5 |
| Self-monitoring technologies (e.g. pulse oximeters and blood pressure monitors at home) | 4.17 | 4 | 4 - 5 |
| Architecture of health information systems | 4.11 | 4 | 4 - 4 |
| Laws pertaining to digital health (e.g. censorship, liability, confidentiality, consent) | 4.11 | 4 | 4 - 4 |
| Personalized medicine | 4.11 | 4 | 4 - 5 |
| Policies and governance of digital health (e.g. national strategies, stakeholders) | 4.11 | 4 | 4 - 4.25 |
| Basic concepts of algorithms (e.g. variable selection, algorithm development, applications) | 4.06 | 4 | 4 - 4 |
| Virtual reality in healthcare (e.g. treatment of mental health disorders, simulations, virtual patient consultations) | 3.67 | 4 | 3 - 4 |
| Customized medical implants for patients | 3.56 | 4 | 3 - 4 |
| Principles of machine learning and neural networks for health applications | 3.56 | 4 | 3 - 4 |
| Principles of virtual clinical trials (e.g. clinical trials run remotely) | 3.56 | 4 | 3 - 4 |
| Image processing and recognition systems | 3.50 | 3.5 | 3 - 4 |
| Interpretation and applications of -omics (e.g. proteomics, genomics, metabolomics) | 3.33 | 4 | 3 - 4 |
| Home drug-delivery systems | 3.11 | 3 | 3 - 3 |
| Biohacking (e.g. citizen and do-it-yourself biology) | 3.06 | 3 | 3 - 3 |
| Mathematical modelling of disease (e.g. disease outbreaks, transmission dynamics) | 3.06 | 3 | 2.25 - 4 |
| Application of chatbots | 2.94 | 3 | 2 - 3 |
| Application of robotics in health (e.g. exoskeletons, bionic arms) | 2.94 | 3 | 2.25 - 3 |
| Principles of computational biology | 2.83 | 3 | 2 - 3.25 |
| Advanced modeling techniques (e.g. bayesian networks, hidden markov models, predictive analytics) | 2.78 | 3 | 2 - 3 |
| 3D reconstruction and printing | 2.61 | 2 | 2 - 3 |
| Speech recognition and natural language processing for health applications | 2.61 | 3 | 2 - 3 |
| Geographic Information System, GIS (capturing and analyzing spatial data) | 2.50 | 2.5 | 2 - 3 |
| Blockchain technologies | 2.11 | 2 | 1 - 3 |
| Principles of nanotechnology | 2.11 | 2 | 1 - 3 |

Table with the average ratings, median values, and interquartile ranges (IQR) of the topics ranked by expert panel for digital health skills

|  | **Average Rating** | **Median** | **IQR** |
| --- | --- | --- | --- |
| Working with clinical decision support systems | 4.89 | 5 | 5 - 5 |
| Using electronic health records in practice | 4.72 | 5 | 5 - 5 |
| Conducting telemedicine in practice | 4.44 | 4 | 4 - 5 |
| Applying digital diagnostic devices | 4.28 | 4 | 4 - 5 |
| Conducting modified physical examinations online | 4.22 | 4 | 4 - 4 |
| Working with ePatients | 4.00 | 4 | 3.25 - 4.25 |
| Basic coding in statistical programs | 3.28 | 3.5 | 3 - 4 |
| Designing health technology | 3.22 | 3 | 3 - 4 |
| Conducting robotic and computer-assisted surgery | 3.17 | 3 | 3 - 3.25 |
| Engaging in digital health entrepreneurship | 2.89 | 3 | 2 - 3.25 |
| Architecting, designing and implementing large-scale health software and apps | 2.22 | 2 | 2 - 3 |

Table with the average ratings, median values, and interquartile ranges (IQR) of the topics ranked by expert panel for digital health attitudes

|  | **Average Rating** | **Median** | **IQR** |
| --- | --- | --- | --- |
| Digital ethics | 4.72 | 5 | 4.25 - 5 |
| Recognition of how digital health impacts the patient-provider relationship | 4.67 | 5 | 4 - 5 |
| Acknowledgement of the advantages and disadvantages of electronic health records | 4.61 | 5 | 4 - 5 |
| Awareness of inequity in access to digital health tools | 4.61 | 5 | 4 - 5 |
| Recognition of algorithm bias | 4.50 | 4.5 | 4 - 5 |
| Appreciation of data privacy and security | 4.44 | 5 | 4 - 5 |
| Importance of data ethics | 4.39 | 4.5 | 4 - 5 |
| Acknowledgement of data overload in digital health | 4.28 | 4 | 4 - 5 |
| Recognizing the importance of interdisciplinary collaboration in digital health | 4.22 | 4 | 4 - 5 |
| Patient empowerment through the use of digital tools | 4.17 | 4 | 4 - 5 |
| Acknowledging the importance of user-centered design and user-friendliness for digital health tools | 4.17 | 4 | 4 - 5 |
| Recognition of the ability to ensure patient compliance through digital health products | 4.06 | 4 | 4 - 5 |
| Appreciating the advantages and disadvantages of consumer genetic testing | 3.89 | 4 | 4 - 4 |
| Considerations in nudging patients with digital health tools | 3.89 | 4 | 4 - 4 |
